# The Need to Analyse Historical Mortality Data to Understand the Causes of Today’s Health Inequalities

**DOI:** 10.3389/ijph.2024.1607739

**Published:** 2024-07-30

**Authors:** Katarina L. Matthes, Kaspar Staub

**Affiliations:** ^1^ Institute of Evolutionary Medicine, University of Zurich, Zurich, Switzerland; ^2^ Swiss School of Public Health SSPH+, Zurich, Switzerland

**Keywords:** population health, historical demography, historical epidemiology, inequality, population-based cohort studies

The past 150 years have seen profound changes in the demography and health of Western populations [1]. Among other things, life expectancy has increased and the causes of death have changed, with the decline of infectious diseases and the rise of non-communicable diseases (NCDs) [2]. Since the early work of John Graunt and William Farr, among others, monitoring causes of death has become an important tool in public health. Changes in causes of death have been well studied, as evidenced by the Global Burden of Diseases studies published since the 1990s [3]. Omran’s epidemiological transition model (1971) and its subsequent modifications are also rooted in this context [4]: The (second) epidemiological transition depicts a decline in mortality from infectious diseases and a shift to degenerative diseases as industrialisation progressed [5, 6]. The last 40 years (the so-called third epidemiological transition), and in particular the recent COVID-19 pandemic, have reinforced the recognition that new and re-emerging infectious diseases pose significant risks; they can spread rapidly around the world and impose a particularly heavy burden in developing countries [7]. However, the patterns of these transitions vary by country, age and sex [8–12].

These fundamental transformations in society have also taken place in Switzerland: Since the end of the 19th century, the standard of living, as measured by GDP *per capita* and real wages, has risen, as has life expectancy, and Switzerland now compares favourably with many other countries [13]. In Switzerland, communicable diseases (especially tuberculosis) were responsible for 22.3% of all deaths in 1901–1905, whereas this proportion had fallen to only 1.1% in 2019 [14]. A few demographers and epidemiologists examined the long-term changes in causes of death in Switzerland in papers published in the 1980s and 1990s: In his cursory review, Guberan showed that mortality from tuberculosis and childhood infections began to decline long before the introduction of immunisation by vaccination and/or the introduction of antibiotics [15]. For the Canton of Geneva, a detailed study of selected causes of death 1901–1980 was carried out by digitising handwritten summary tables [16]. The limitations and improving quality of such data sources on causes of death were also discussed (validity of the determination of causes of death, revisions of classification systems, etc.) [16]. However, the last 2-3 decades and especially the last few years of the development of causes of death under the impact of the COVID-19 pandemic have not yet been placed in the longer-term historical context.

**FIGURE 1:**
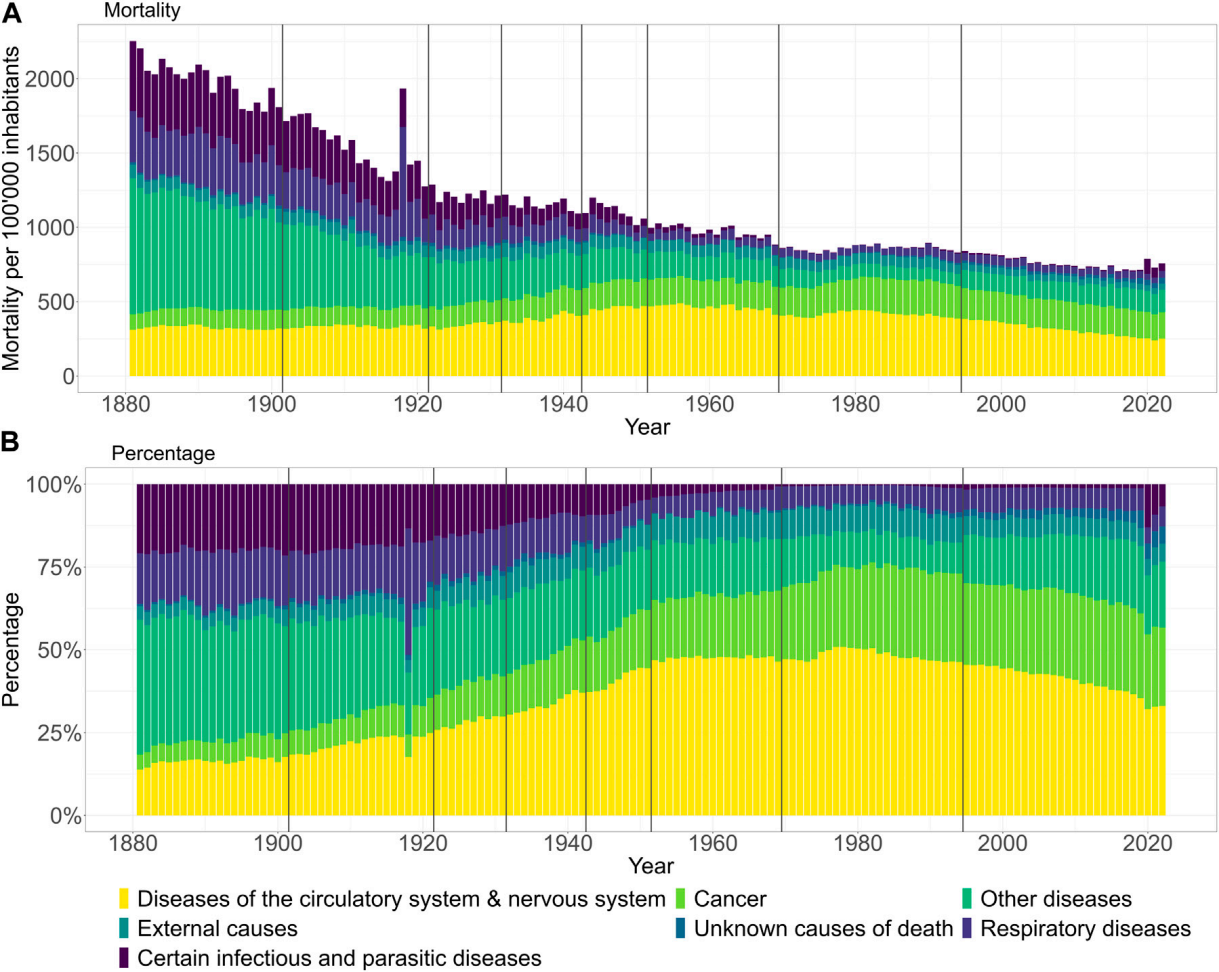
Changes in the major categories of primary causes of death in Switzerland at the population level since 1881: **(A)** mortality per 100,000 inhabitants and **(B)** relative figures in percentage. Vertical grey lines indicate updates of causes of deaths classifications. (Switzerland 1881–2022).

The primary main causes of death available for the years from 1881 to 2022 are shown in Figure 1 using data from the Historical Statistics of Switzerland (HSSO) [17] for the years 1881–1968 and the cause of death statistics of the Swiss Federal Statistical Office (FSO) for the years 1969–2022 [14]. From 1877 (when an official catalogue of causes of death was first introduced for Switzerland) to the present day, there have been several changes in the official classifications of causes of death (1901, 1921, 1931, 1942, 1951, 1969, and 1994). Until 1969, Switzerland used its own system of causes of death, which was revised after census years. In 1969, Switzerland adopted the international ICD codes (ICD-8 until 1994, then ICD-10). The conversions between the catalogues are well documented, allowing homogeneous series to be constructed.

In Figure 1, the long-term changes and certain discontinuities in the changes in the primary causes of death can be tracked at the population level both in mortality per 100’000 inhabitants (A) and relative in percentages (B) terms using the large categories predefined according to FSO. In Figure 1A, the general reduction in mortality over time is obvious. The pandemic year 1918 stands out clearly, as does the pandemic year 2020 relative to the steady figures since around 1980, albeit much less markedly. In both of these years, the increase in deaths can mostly be attributed to the major cause categories “Infectious and parasitic diseases” and/or “Diseases of the respiratory system.” In Figure 1B, however, the proportion of mortality attributed to “Infectious and parasitic diseases” and “Diseases of the respiratory system” have declined steadily, while the disease category “Circulatory system & nervous system” rose until the 1960s and has been declining again since 1990. The proportion of cancer mortality has also increased over the years and remained stable since 1990. In relative terms, the two pandemic years 1918 and 2020, represent obvious disruptions in these proportional trends due to a temporary and sudden increase in mortality from infectious diseases and respiratory illnesses. While the magnitude of the impact of the 1918 influenza pandemic outbreak is well documented and known, our superficial visualization underlines the historical dimension of the COVID-19 pandemic from a different perspective. Until now, it was simply known that all-cause excess mortality reached levels not seen since the 1918–1920 pandemic, especially in autumn 2020 in the context of the second COVID-19 wave.

Despite the crisis events of recent years, the progress made in reducing mortality over the last 150 years is remarkable, and these accomplishments in the health and public health sector are often forgotten today. However, it should be noted that mortality and changes in the causes of death only reflect part of the burden of disease and the state of health of a population, and that a more detailed and broader view is needed to obtain a comprehensive picture. Trends at the aggregated population level often hide essential socio-demographic, socio-economic and regional differences that need to be monitored and considered. Socio-demographic disparities persist, and the advancements in health achieved over the last 200 years have not been equally distributed among all individuals. Regarding causes of death and socio-demographic factors in recent years, most of the publications in Switzerland took place as part of the Swiss National Cohort Study and highlighted socioeconomic and demographic inequalities in mortality [18]. COVID-19 has once again highlighted health inequalities worldwide [19, 20]. Individuals previously vulnerable to the social determinants of health were also more adversely affected by COVID-19 [21], which also led to mortality disparities in Switzerland during the pandemic [22].

At present, understanding of the historical roots, causes and drivers of these historical health inequalities is limited. It is still unclear when and how this health gap developed, and whether it remains constant or even increases. Addressing today’s health inequalities and public health challenges require long-term population-based cohort studies. Particularly in Switzerland, where a large population-based cohort has not yet been established, there is a lack of representative data on relevant health outcomes and determinants [23]. In the absence of such data, it will remain unable to comprehensively address the intricate socio-economic dynamics and their impact on health inequities. However, these issues go beyond Switzerland and affect many Western countries. That’s why international initiatives like the EU COST Action “CA22116 - The Great Leap. Multidisciplinary approaches to health inequalities, 1800–2022” [24] are so important, which address precisely these longer-term transitions from the perspective of causes of death. The focus is on improving health equity, and this includes continued monitoring of inequalities, which is better achieved with historical context and a better understanding of their roots and drivers. While initiatives such as this COST Action significantly improve our understanding of the causes of health inequalities, they alone are not sufficient to address the public health challenges of the 21st century - it is essential to establish additional long-term population-based cohorts.
